# Algoneurodystrophy in a Patient with Major Depressive Disorder

**DOI:** 10.1155/2021/9981521

**Published:** 2021-10-09

**Authors:** Virgínia Henriques, Sérgio Henriques

**Affiliations:** ^1^Hospital Garcia de Orta, Department of Psychiatry, Av. Torrado da Silva, 2805-267 Almada, Portugal; ^2^Hospital Central do Funchal, Department of Psychiatry, Av. Luís de Camões, No. 57, 9004-514 Funchal, Portugal

## Abstract

*Introduction*. Not infrequently, in patients with a psychiatric illness who have concomitant physical symptoms, these symptoms are often wrongly attributed to a psychiatric illness. Consequently, there is a delay in establishing the correct diagnosis, which may have an impact on the prognosis of the disease. The authors aim to present a case report of a patient with a diagnosis of major depressive disorder and conversion disorder that was later correctly diagnosed with algoneurodystrophy. The authors intend to draw attention to the importance of a careful medical history and this entity. *Case Presentation*. A patient went to the emergency department multiple times with complaints of decreased strength and pain in the right upper limb, concomitantly with depressive symptoms. The patient was first diagnosed with conversion disorder and major depressive disorder. After the worsening of the clinical condition with the appearance of neuropathic pain and the exclusion of other organic pathologies, the probable diagnosis of algoneurodystrophy was made. At that time, the patient started treatment and a favorable clinical evolution was observed. *Discussion*. The clinical case highlights the importance of conducting a careful medical history in a patient with a psychiatric illness, so as not to mistakenly exclude the presence of an organic disease. The absence or delay in making a correct diagnosis can have adverse consequences in terms of the prognosis of the disease.

## 1. Introduction

Not infrequently, in patients with a psychiatric illness who have concomitant physical symptoms, these symptoms are often wrongly attributed to a psychiatric illness [1]. Consequently, there is a delay in establishing the correct diagnosis, which may have an impact on the prognosis of the disease.

The authors aim to describe a case report of a forty-five-year-old woman with a diagnosis of major depressive disorder and a diagnosis of conversion disorder that was later correctly diagnosed as algoneurodystrophy. The authors intend to draw attention to the importance of doing a careful medical history and recognizing this entity that can go unnoticed.

Algoneurodystrophy or type I complex regional pain syndrome is a rare and underdiagnosed clinical disorder. It is a form of chronic pain that usually affects an arm or a leg, and its cause is not clearly understood [2]. Data on its incidence are scarce, and statistics vary between 5.45 per 100,000 person-years in the USA and 26.2 per 100,000 person-years in Europe, with a predilection for females in the proportion of 3 : 1 [2]. It is most common in the age range between 37 and 53 years [2–4].

Clinical diagnosis is based on typical signs localized in an extremity without an identifiable nervous lesion: allodynia, hyperalgesia, pseudoparalysis, swelling, changes in skin vascularization, and sweating. There appears to be an increased preponderance for the upper limbs with a ratio of 3 : 2 compared to the lower limbs [2]. It is mostly associated with a history of previous trauma, namely, fractures. Surgical interventions, local inflammatory processes, minimally invasive procedures, and pathologies that progress with immobility constitute other risk factors for their appearance [3]. However, it can appear spontaneously, in the absence of an identifiable precipitating event. In the early clinical presentation, it may be necessary to perform differential diagnoses with various pathologies like rheumatic, orthopedic, infectious, vascular, and psychosomatic disorder (conversion disorder).

## 2. Case Presentation

D. is a 45-year-old Caucasian woman, native, and resident of the Autonomous Region of Madeira (Portugal). In terms of personal medical history, there is a history of major depressive disorder. She is medicated with the following therapeutic regimen: venlafaxine 75 mg, mirtazapine 30 mg, lorazepam 1 mg, and quetiapine 100 mg. The patient has no other pathologies.

The patient went to the emergency department of the Central Hospital of Funchal with a clinical condition characterized by depressed mood, anhedonia, clinophilia, and complaints of decreased strength in the right upper limb for two weeks. She reported that she had an episode of syncope and fall, and after that event, complaints of decreased strength in the right upper limb started. The patient denied the occurrence of head trauma after the fall. In the emergency department, a physical examination and a summary neurological examination were performed, which were unremarkable. Regarding the examination of mental status, the patient had a depressed mood, without other changes. The patient underwent a brain CT scan that showed no changes. D. was discharged from the emergency room with a diagnosis of major depressive disorder, and a therapeutic adjustment was made with an increase in venlafaxine to 150 mg per day.

A few days later, D. went again to the emergency department because she had the same complaints of decreased strength in the right upper limb, without any clinical improvement. The physical examination was unremarkable. The neurological examination revealed decreased sensitivity and pseudoparalysis of the right upper limb. Complementary diagnostic tests were performed that did not show alterations, namely, complete blood count, biochemistry with an assessment of renal function, liver parameters, sedimentation speed, and C-reactive protein. D. was discharged from the hospital with a diagnosis of conversion disorder and was instructed to maintain the usual therapy.

A week later, the clinical condition worsened, without an identifiable precipitating event, and the patient went again to the emergency department. She presented pseudoparalysis of the right upper limb, intense pain, redness, swelling, and functional impairment. She did not present clinical or radiologic evidence of fracture or dislocation of the right upper limb. Diagnostic tests were performed: X-ray of the right upper limb did not reveal a fracture but showed signs of bone demineralization, and the CT scan of the cervical spine was normal. Taking into account the clinical presentation, the probable diagnosis of algoneurodystrophy was placed. Rehabilitation treatment was started, as well as treatment with ibuprofen 1800 mg at 2400 mg/day. The clinical evolution was favorable, with progressive functional recovery.

## 3. Discussion

The exact pathophysiological mechanisms that are at the origin of algoneurodystrophy are still unknown. By definition, there is no organic damage to the nervous system; however, there seems to be an exacerbation of normal tissue reaction mechanisms in the face of a painful stimulus [4]. The proposed pathophysiology is based on the creation of an anomalous reflex nervous arch, which spreads from the periphery to the cerebral cortex with subsequent efferent transmission to the spinal cord. From this, an impulse from the sympathetic nervous system appears, which results in hyperstimulation of peripheral sympathetic activity, with a corresponding neurovascular inflammatory response, which causes pain [4, 5]. Immobilization can perpetuate the reflex arc, and its avoidance is crucial to interrupting this circuit.

It appears that psychological factors play a role in this entity, but the exact relationship is unknown [6, 7]. Several studies have indicated that chronic pain predicts higher depression, anxiety, and anger, and they postulate that pain influences psychological symptoms which in turn exacerbate pain symptoms [7]. However, there is no indication that psychological factors cause the onset of pain, autonomic dysfunction, and movement disorders in patients with algoneurodystrophy [7].

In the case of this patient, it was not easy to diagnose the pathology at the beginning. The clinical condition was not present in its typical form at the initial evaluation, since the patient only presented complaints of decreased strength in the right upper limb, concomitantly with depressive symptoms. However, the patient had a risk factor for algoneurodystrophy, namely, the history of trauma. Consequently, there was a delay in the assignment of the correct diagnosis, and instead of that, the diagnosis of conversion disorder was attributed. Afterward, the patient showed signs compatible with neuropathic pain, namely, severe pain, disproportionate to stimuli, in a location that does not correspond to a dermatome; redness; swelling; and pseudoparalysis. Given this clinical presentation, the probable diagnosis of algoneurodystrophy was then placed.

Although the diagnosis is clinical, some complementary means of diagnosis can be useful in excluding other pathologies, namely, rheumatic, orthopedic, infectious, vascular, and psychosomatic disorder—conversion disorder [5]. Algoneurodystrophy can be difficult to diagnose, as can be seen by analyzing this clinical case. In an initial presentation, it is possible to confuse it with conversion disorder as a nerve injury is not identified, the pain is disproportionate in magnitude and duration to the precipitating event, and the functional impairment exceeds what is expected [6].

In analytical terms, there are no significant changes in algoneurodystrophy. Radiography may show bone demineralization, but this radiological alteration is only present in the late stage of the disease, which is a disadvantage for its use [4, 5]. In this case, several complementary diagnostic tests were requested that did not reveal changes, namely, laboratory tests, a CT scan of the brain, and a CT scan of the cervical spine. However, the patient showed signs of demineralization of the bone in the radiography.

Because its pathophysiology is incompletely understood, there are no guidelines for its management. Two major approaches to the treatment of early algoneurodystrophy are pain relief (anti-inflammatory therapy) and physical rehabilitation [5]. Early diagnosis and multidisciplinary treatment are essential to avoid sequels or evolution to chronicity [5–7].

This case report highlights the importance of conducting a careful clinical history since a high degree of suspicion is required to diagnose algoneurodystrophy. We also mention the importance of not excluding physical symptoms in a patient with a mental illness, as they may be due to an organic pathology, and for that reason, they must be investigated. When a patient with algoneurodystrophy is not diagnosed and instead is wrongly attributed to a psychiatric pathology, negative consequences can arise, since the patient will not have rapid access to treatment interventions that could improve quality of life and daily functioning and thwart disease progression.

We emphasize that more studies are needed in this area to better understand this entity. With increased knowledge, we believe that both physicians and mental health professionals can better diagnose, treat, and manage algoneurodystrophy symptomatology.
